# Cocaine addiction severity exacerbates the negative association of lifetime lead exposure with blood pressure levels: Evidence from a pilot study

**DOI:** 10.4103/ed.ed_21_19

**Published:** 2019-09-27

**Authors:** Elena Colicino, Danielle B. Hazeltine, Kelly M. Schneider, Anna Zilverstand, Keren Bachi, Nelly Alia-Klein, Rita Z. Goldstein, Andy C. Todd, Megan K. Horton

**Affiliations:** 1 Department of Environmental Medicine and Public Health, New York, NY, USA; 2 Department of Psychiatry, New York, NY, USA; 3 Department of Psychiatry; Department of Neuroscience Icahn School of Medicine at Mount Sinai, New York, NY, USA

**Keywords:** Blood pressure, cardiovascular health, cocaine use, K-shell X-ray fluorescence, lead exposure

## Abstract

**Background:**

High blood pressure (BP) is associated independently with cocaine use and lead exposure. It is not known whether cocaine use and lead exposure act jointly to disrupt cardiovascular health.

**Objective:**

To determine whether cocaine use modifies the association between cumulative lead levels and elevated BP.

**Materials and Methods:**

We measured cumulative tibia lead levels in 35 adults: 20 with cocaine use disorder (CUD) and 15 non-CUD controls using *in vivo* K-shell X-ray fluorescence. Generalized estimating equation regression determined associations between log_2_-transformed lead and BP (systolic, diastolic, and mean arterial pressure) and assessed the modifying association of cocaine use (as addiction severity) on the lead-BP relationship, adjusting for age, sex, smoking, and education. Sensitivity analyses included correction for potential selection bias.

**Results:**

Cases and controls differed by sex (%male: 90% vs. 67%), age (50.7 vs. 39.9 years), education (12.8 vs. 14.4 years), and tibia lead (3.50 vs. 2.35 μg/g). Lead was positively associated with systolic (*P* = 0.01) and diastolic BP (*P* = 0.01). We observed an interaction between lead and addiction severity on BP (*P* values for systolic BP: 0.01, diastolic BP: 0.003, and mean arterial BP: <0.0001); the association was stronger among individuals with more severe cocaine addiction: Systolic BP: Est.: 17.89, 95% confidence interval (CI): 9.52; 26.26, diastolic BP Est.: 17.89, 95% CI: 7.33; 13.79, mean arterial BP: Est.: 13.09, 95% CI: 10.34; 15.83.

**Conclusions:**

Lead was adversely associated with BP. This association was strongest among individuals with more severe cocaine addiction. The results from this small pilot study suggest that the interaction between lead and cocaine should be considered in studies of substance abuse-related health outcomes.

## INTRODUCTION

High blood pressure (BP), defined as elevated systolic or diastolic BP levels, affects 78 million US adults (one in three) and is the leading factor worldwide for heart diseases, stroke, kidney disease, and mortality.^[1]^ High BP is a complex trait resulting from interactions of multiple genetic and environmental or lifestyle factors.^[2–5]^ Both cocaine use and lifetime lead exposure have been independently associated with increased BP;^[6,7]^ the potential interaction of these exposures on cardiac health is unknown.

Lead is a well-studied, central nervous system toxicant, exposure to which has been associated with adverse neurologic outcomes in adults and children.^[8,9]^ Cumulative lead exposure is also associated with elevated BP and hypertension.^[3–5]^ Since banning leaded gasoline, paint, and solder and passing industrial regulations and restrictions on its use, lead exposure has declined significantly in the United States.^[10]^ Exposure persists through occupational exposures and in socioeconomically disadvantaged communities.^[11]^

In adults, over 95% of the body burden of lead is stored in the skeleton. ^[12,13]^ Bone resorption transfers lead to blood and soft tissues and can be a main source of exposure and toxicity. ^[14,15]^ Tibia lead concentration, measured using K-shell X-ray fluorescence (KXRF), is a well-established biomarker of cumulative lead exposure, with a half-life of over 30 years. ^[16,17]^ In epidemiologic studies, bone lead has been associated with elevated BP. ^[18]^

In the recent decade, the number of regular cocaine users is increasing, and the Drug Enforcement Administration recently estimated that 1.7 million U.S. adults regularly use cocaine.^[19]^ In cohort and laboratory studies, regular cocaine consumption, defined as years of continuous use, is one of the lifestyle factors associated with arterial vasoconstriction and increased BP.^[6,18,20]^ Some studies have also suggested that regular cocaine use may play a central role in the development of heart diseases and may be a precursor of chronic, long-term consequences, including high BP levels.^[7,20]^

The goal of this study was to examine whether cumulative lead exposure and cocaine use jointly contribute to elevated BP. We hypothesized that cocaine addiction severity modulated the association of cumulative lead exposure on BP levels, with stronger associations for cases more severely addicted. To determine those associations, we leveraged an ongoing case–control study of cocaine users and nonusing controls. ^[21]^

## MATERIALS AND METHODS

### Study population

Participants included 20 nontreatment-seeking individuals with cocaine use disorder (CUD) and 15 healthy (noncocaine using) controls recruited from New York City, who undergo clinical diagnostic interviews and physical examinations.^[22]^ Between 2015 and 2017, we recruited 20 cases with CUD and 15 healthy controls with existing data on BP, cocaine use, and relevant covariates – age, sex, education (years of school), body mass index, and smoking status (ever/never) – to participate in an additional study of tibia lead concentrations. Eight participants underwent clinical diagnostic interviews and physical examinations twice, approximately 2 years apart. For the second visit, tibia bone lead concentrations were imputed to increase the sample size. All participants provided written informed consent as per the institutional review board.

### Blood pressure levels

To assess BP, a trained staff member measured systolic and diastolic BP levels on the dominant arm of each participant with a semiautomatic, digital manometer when they were seated. When the levels of systolic or diastolic BP were over 140 or 90 mmHg, respectively, the study staff validated the readings on the other arm within 20 min. The standard calculation for mean arterial pressure (MAP; the average pressure in a participant’s arteries during one cardiac cycle) is described elsewhere. ^[23]^

### Cocaine use

Information on years of regular cocaine use and number of drugs regularly used was collected by trained personnel, supervised by a licensed clinical psychologist, via the Structured Clinical Interview for Diagnostic and Statistical Manual of Mental Disorders, Fifth Edition for Axis I disorders. ^[21]^ We categorized years of regular cocaine use and number of drugs regularly used to facilitate the interpretation of the analyses. Cocaine addiction severity was the quotient of years of regular cocaine use and age (in years). All the controls reported 0 years of cocaine use (C0) and were the reference group for all analyses. Among CUD cases, we dichotomized cocaine addiction severity at the 3^rd^ quartile: C1 = lowest 75% of use (>0%–44% of lifetime) and C2 = highest 25% of use (44%–68% of lifetime). We defined multiple drug dependence as the number of drugs regularly used, which was categorized to discriminate between addiction to cocaine only (D1) and addiction to multiple drugs (D2), absence of drug addiction (D0) served as the reference. Information on multiple drug addiction was available for 16 CUD and 10 healthy participants.

### Tibia lead concentrations

Tibia lead was measured via a KXRF method previously described. ^[16,17]^ Briefly, participants sat in a lead-free chair for 30 min while midtibial diaphysis lead atoms were fluoresced using ^109^Cd. Concentrations were reported as **μ**g of lead per gram of bone mineral and included a measure of uncertainty. ^[8,16]^ One control with a nondetectable (negative) tibia lead concentration was excluded from the analyses because it was an outlier, leaving a total of 20 cases and 15 controls.

### Statistical analysis

The main analysis included 35 individuals, 8 of whom had BP and covariate information collected at two visits which occurred 2 years apart, on average (a period of time over which tibia lead does not change measurably^[24]^), allowing the imputation of missing tibia lead concentrations for the second visit. For hypothesis testing, we log_2_-transformed the tibia lead concentrations. BP variables were normally distributed and treated as continuous.

To determine the association between each outcome (systolic, diastolic, and MAP levels) with tibia lead concentrations and cocaine addiction severity (categorized), we used generalized linear regression models with generalized estimating equations with an exchangeable working correlation structure and an empirical variance estimate to account for repeated measures within participants:

$$Y_{\text{it}}\;=\;b_{0}\;+\;b_{1}X_{1\text{it}}\;+\;\ldots \;+\;b_{\text{p}}X_{\text{pit}}\;+\;\beta_{1}{\text{Lead}}_{\text{it}}\;+\;\beta_{2}{\;\text{C1}}_{\text{it}}\;+\;\beta_{3}\;\text{C}2_{\text{it}}\;+\;\varepsilon_{\text{it}},$$

in which *Y*_it_ was the outcome (systolic, diastolic, and MAP levels) of the i^th^ case at t^th^ time, b_0_ was the overall intercept, *X*_1it_–*X*_pit_ were the covariates included, and β_1_, β_2_, and β_3_ were the associations between the outcome with tibia lead (lead) and cocaine use (cocaine addiction severity or multiple drug dependence).

To assess whether cocaine use modified the association between tibia lead levels and BP, we included interaction terms between tibia lead levels and each category of cocaine addiction severity (C0, C1, and C2) in the previous model. We also determined the significance of the trend of this association by including cocaine addiction severity as a single continuous variable.

As a secondary analysis, we evaluated whether tibia lead-BP associations were modulated by multiple drug dependence, discriminating between those participants dependent on cocaine only (D1) and those on multiple drugs (D2).

All analyses were adjusted for sex (male/female), age at the time of the visit (years, continuous), smoking status (ever/never), and education (years, continuous). Covariate selection was based on previous studies and associations with the outcomes. ^[25,26]^

Body mass index (BMI), missing for eight participants, has previously been shown to be associated with higher BP levels. ^[27]^ We conducted multivariate linear regressions as sensitivity analyses, adjusting for BMI as a continuous variable, and restricted our sample to the visits at which tibia lead was measured. Demographic differences between CUD and healthy participants may influence the results. To reduce any possibility of selection bias, we conducted a sensitivity analysis using the inverse probability weighting technique. Weights were computed as the inverse of a probability, defined by a logistic regression in which the dependent variable was the case status (being or not a CUD participant) and the independent variables were the characteristics with significant differences between the two groups. Due to the unbalance between men and women in our sample, we also conducted a sensitivity analysis only on men. The results are presented in Supplementary Data.

## RESULTS

### Sample characteristics

CUD cases were significantly older (mean [years] ± standard deviation [SD]: 50.79 ± 5.92 vs. 39.09 ± 10.17, *P* < 0.01) and less educated [mean years of education ± SD: 12.80 ± 1.70 vs. 14.73 ± 3.52, *P* = 0.04; Table 1] than healthy controls. Compared to healthy controls, more CUD cases were smokers [95% vs. 33%, *P* < 0.01; Table 1]. Tibia lead levels were higher in CUD cases than that in the healthy controls [mean ± SD: 3.50 ± 0.88 vs. 2.35 ± 1.51, *P* < 0.01; Table 1]. There were no significant differences between groups in the other covariates or BP levels.

### Main associations of cocaine use and tibia lead on blood pressure

An average two-fold increase in tibia lead was associated with significantly increased systolic (Est.: 7.06; 95% confidence interval [CI]: 1.7–12.42) and diastolic (Est.: 4.76; 95% CI: 1.13–8.39) BP measures [Figure S1 and Table S1]. In general, cocaine addiction severity was not independently associated with higher BP [Table S1]. Among the most severely addicted group (C2), cocaine addiction severity was significantly associated with lower systolic BP (Est.: −13.10; 95% CI: −26.07, −0.13). However, the Wald test for cocaine addiction severity showed no statistical significance in the model with systolic BP (*P* = 0.72).

### Modifying association of cocaine addiction severity on the relationship between tibia lead and blood pressure

**Figure 1:**
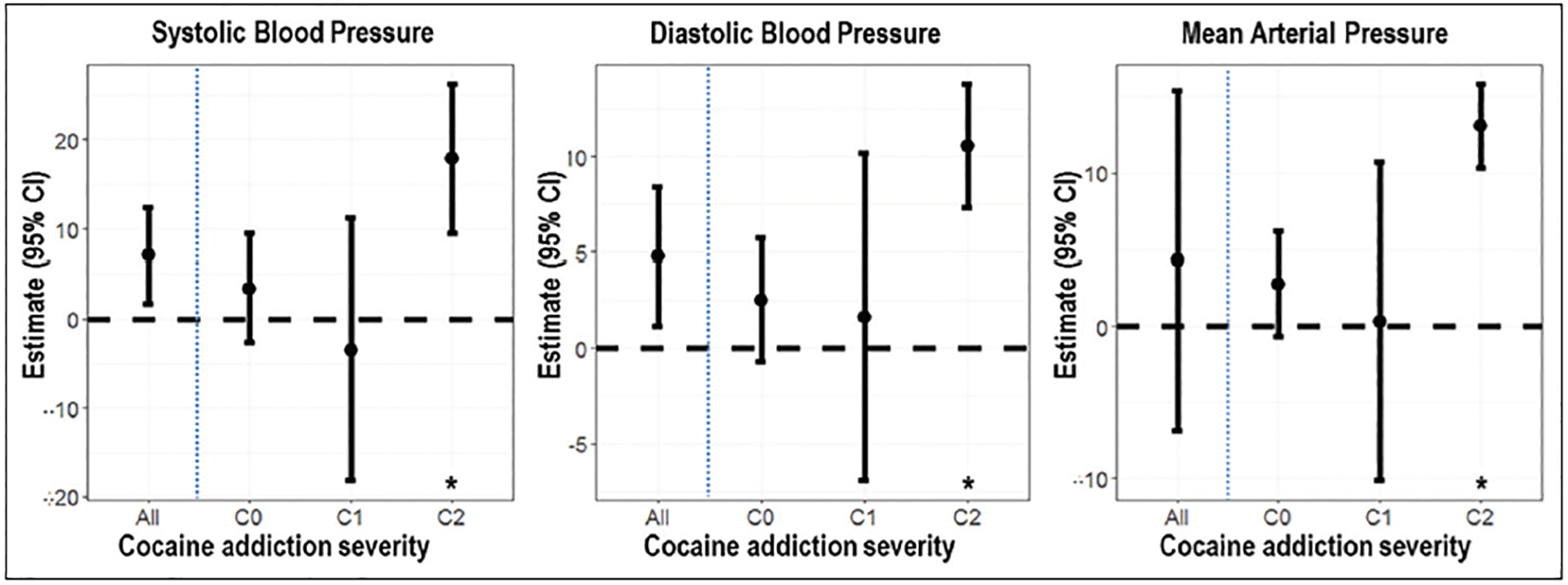
Tibia lead-blood pressure relationship and cocaine addiction severity as modifier of the relationship. Main effect of (log2-transformed) lifetime lead exposure on blood pressure levels and modifying effect of cocaine addiction severity on the relationship between bone lead (log2-transformed μg/g bone) and blood pressure levels. Cocaine addiction severity categories include all participants (All: main effect); nonusers (C0, reference), <44% lifetime use years (C1), >44% lifetime use (C2: square). 95% CI: 95% confidence interval; *P for interaction <0.05

Tibia lead was significantly associated with higher systolic, diastolic, and MAP in CUD cases reporting the most severe cocaine addiction (Est.: 17.89; 95% CI: 9.52–26.26 for systolic BP; Est.: 10.56; 95% CI: 7.33–13.79 for diastolic BP; and Est.: 13.09; 95% CI: 10.34–15.83 for MAP), compared to individuals with no cocaine use or in individuals with less severe cocaine addiction [Figures 1, S2 and Table S2]. The interaction between cocaine addiction severity and bone lead on BP was significant for users reporting the most severe cocaine addiction (*P* < 0.001 for systolic, diastolic, and MAP) [Figures 1, S2 and Table S2].

### Modifying association of multiple drug dependence on the relationship between tibia lead and blood pressure (secondary analysis)

Multiple drug dependence was positively associated with cocaine addiction severity (Pearson’s coefficient = 0.506; *P* = 0.002). Multiple drug dependence modified the relationship between tibia lead concentrations and systolic BP and MAP [interaction = 0.064 and 0.012, respectively, Table S3], with stronger associations among individuals dependent on multiple drugs [Est.: 14.57; 95% CI: 4.48–24.66 for systolic BP and Est.: 9.32; 95% CI: 6.46–12.18 for MAP; Table S3] than in healthy controls or individuals using only cocaine.

### Sensitivity analyses

We restricted our sensitivity analyses to 26 individuals with BMI and excluded repeated measures. The results confirm the positive and significant association between a two-fold increase in tibia lead and diastolic BP (Est.: 4.87; 95% CI: 0.72–9.02) [Table S4]. In these analyses, we did not observe a significant modifying effect of cocaine addiction severity on the relationship between tibia lead and any of the BP outcomes. This may be due to the limited number of individuals with severe cocaine addiction (C2) in this subset [Tables S5]. The results corrected for weights and in men only were consistent in magnitude and significance with main findings [Tables S6–S9].

## DISCUSSION

The findings from this case–control study confirmed that tibia lead exposure was positively associated with BP levels. We also showed that the severity of cocaine addiction modulated the association of tibia lead exposure on systolic BP levels, to greater effect in individuals with more severe cocaine addiction. We further demonstrated that multiple drug dependence (number of different types of drugs regularly used) modified the relationship between tibia lead and both systolic BP and MAP, with more adverse associations for individuals dependent on multiple drugs. All our findings were controlled for sociodemographic and lifestyle factors. These results suggest that the potential adverse interaction between lead and drug use (i.e., cocaine) should be considered in studies of substance abuse-related health outcomes.

In this study, we measured tibia lead levels which have been shown to be a better biomarker than blood lead to capture the cumulative lead exposure and evaluate its long-term health effects in epidemiology studies. ^[28]^ Further, tibia lead, compared to patella lead, is considered a biomarker of cumulative lead dose. ^[29,30]^ Tibia lead levels are acquired using a highly specialized technique (KXRF) available in few centers worldwide. Despite steady declines in U.S. lead levels in response to the banning of leaded gasoline, paint, and solder and the passing of industrial regulations and restrictions on its use, the U.S. Occupational Safety and Health Administration showed that lead exposure is still a public health concern, estimating that more than 1.6 million employees have been exposed to lead on an annual basis^[31]^ and more than 2000 U.S. adults suffering from elevated persistent lead levels in 2012.^[32]^ Previous studies associating tibia bone lead levels with BP showed higher cumulative lead levels than the CUD cases and non-CUD controls included in our study.^[18]^ This study supports the evidence that lead exposure is still traceable in active adults and the interaction of lead with novel exposures or lifestyle factors might exacerbate health outcomes.

Previous research has found that tibia lead exposure and cocaine use independently increase BP; our novel research suggests that they may act jointly to disrupt cardiovascular health. Elevated systolic BP has been consistently and independently associated with both bone lead exposure and cocaine use. ^[3,7,14,20]^ The findings of the interplay among regular cocaine use, tibia lead exposure, and systolic BP are particularly interesting, given that systolic BP is directly and continuously related to the risk of cardiovascular disease.^[2]^ Consistent with the literature, this study shows that diastolic BP was not consistently associated with bone lead or cocaine use.^[7,18]^ One reason may be that diastolic BP increases with age until 55 years, then subsequently declines.^[2,33]^ This relationship between diastolic BP and age led to elevated diastolic BP only in the young and middle-aged populations, rather than in the elderly.^[34]^

Our population study consisted mostly of men, the majority of whom reported low levels of education, thus limiting the generalizability of our results. Further, in our pilot, CUD cases were older than non-CUD controls. Despite the lack of generalizability, it is possible that CUD cases reflected in this study may represent the population most vulnerable to adverse health effects of a lead–cocaine interaction. While our analyses showing a lead–cocaine interactive relationship controlled for a number of potential confounders, some residual confounding is possible. For example, the relationship may be confounded by adverse neurologic outcomes of lead exposure on mental health and cocaine addiction. ^[35]^ Physical activity is a known predictor of cardiac health and BP^[36]^ and another possible confounder of the observed relationships. Unfortunately, we do not have information on physical activity in this cohort.

Due to the limitations of a pilot study including the small sample size and the limited lead biomarkers available, further analyses, including an increased number of participants, the addition of patella lead and concurrent blood lead levels, additional information on other potential exposures (i.e., other illicit and prescriptive drug use), and information on physical activity, are needed to confirm our hypothesis that cocaine use modifies the adverse association between lead and BP. Cohort studies focusing on collecting substance abuse information should consider evaluating the interaction between substance abuse and environmental factors in relation with the outcomes of interest.

## CONCLUSION

Cumulative (tibia) lead levels were associated with increased levels of systolic and diastolic BP. The detrimental association between lead and BP levels was most pronounced among participants with more severe cocaine addiction or with multidrug addition, compared to all other individuals.

## Supplementary Material

1

## Figures and Tables

**Table 1:** Study characteristics: Sociodemographic characteristics, blood pressure, and tibia lead concentrations for cocaine use disorder cases and controls included in this study (*n*=35)

Characteristic	CUD (*n*=20)	Healthy participants (*n*=15)	*P*
Age, mean±SD	50.79±5.92	39.09±10.17	<0.01*
Sex, males, *n* (%)	18 (90)	10 (66.7)	0.11
Education (years)	12.80±1.70	14.73±3.52	0.04*
BMI (kg/m^2^)^†^	27.16±3.64	27.97±5.39	0.65
Smoking^‡^, *n* (%)	19 (95)	5 (33.3)	<0.01*
Cocaine use (overall years), mean±SD	21.10±9.45	0	<0.01*
BP (mmHg), mean±SD			
Systolic	129.00±20.52	125.80±17.99	0.63
Diastolic	81.50±11.01	78.07±8.41	0.32
MAP	97.33±13.25	93.98±10.65	0.43
Tibia lead (Pb)^§^, μg/g bone	3.50±0.88	2.35±1.51	0.01*

* *P*<0.05,

† BMI, missing values; cases (3), healthy participants (5),

‡ Number of subjects who have ever smoked, self-reported,

§ Lead (Pb) levels were log_2_-transformed.

*P*-levels for the characteristics age, education, BMI, MAP, cocaine use, BP, and Pb were found using independent samples *t*-tests, *P*-levels for sex, BMI missing, and smoking were found using Chi-square test. BMI: Body mass index, MAP: Mean arterial pressure, CUD: Cocaine use disorder, BP: Blood pressure
