# Supplementary material for: Cocaine addiction severity exacerbates the negative association of lifetime lead exposure with blood pressure levels: Evidence from a pilot study
Source: Environ Dis. Author manuscript; Available in PMC 2021 Jan 22. (PMC7822570; doi:10.4103/ed.ed_21_19)
Supplement: 1 [file NIHMS1067879-supplement-1.pdf]

[Downloaded free from http://www.environmentmed.org on Monday, October 14, 2019, IP: 146.203.130.213]

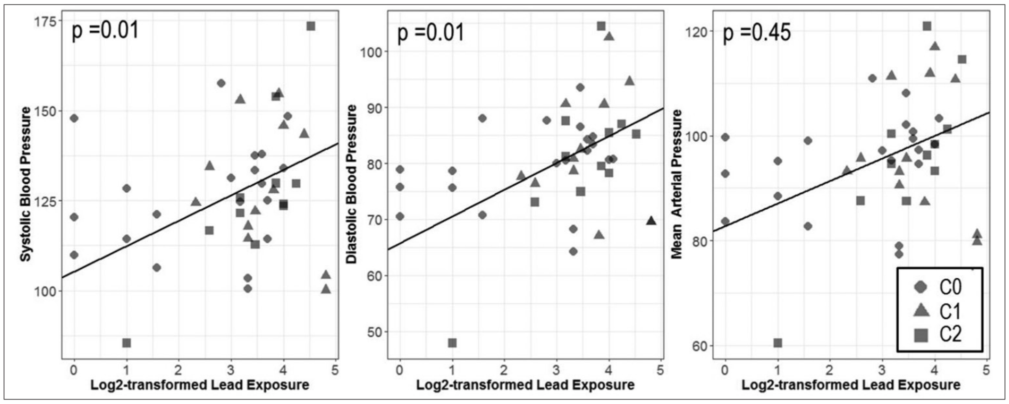

Figure S1: Significant main effect of (log$_2$-transformed) lifetime lead exposure (X-axis) on blood pressure levels (Y-axis). Each dot represents a single individual, and shape coding represents the cocaine addiction severity: nonusers (C0: circle), <44% lifetime use years (C1: triangle), >44% lifetime use (C2: square). *P* value for the association using generalized estimating equation

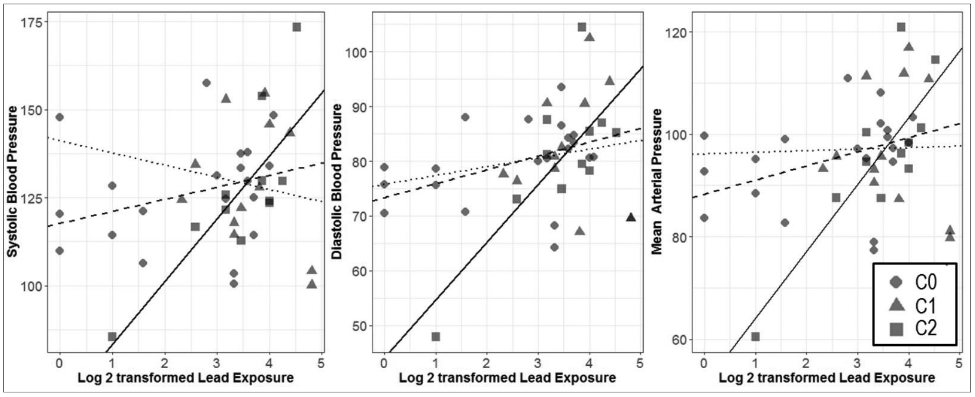

Figure S2: Modifying effect of cocaine addiction severity on the relationship between bone lead (log$_2$-transformed μg/g lead) and blood pressure. Each dot represents a single individual, and shape coding represents the cocaine addiction severity: nonusers (C0: circle, dashed line), <44% lifetime use years (C1: triangle, dashed line), >44% lifetime use (C2: square, solid line)

[Downloaded free from http://www.environmentmed.org on Monday, October 14, 2019, IP: 146.203.130.213]

Table S1: Main effect of bone lead concentrations (log$_2$ transformed ug/g bone) or cocaine addiction severity* on blood pressure levels

Variable	Category	*n*	Systolic BP			Diastolic BP			MAP		
			β	95% CI	*P*	β	95% CI	*P*	Β	95% CI	*P*
Lead	mg/g bone	35	7.06	(1.7 ;12.42)	0.01*	4.76	(1.13 ;8.39)	0.01*	4.29	(-6.84 ;15.42)	0.45
No cocaine use	C0	15	Ref.	.	.	Ref.	.	.	Ref.	.	.
<3rd quartile cocaine addiction severity	C1	11	2.37	(-12.34;17.09)	0.75	1.92	(-8.31 ;12.16)	0.71	0.12	(-29.15 ;29.39)	0.99
>3rd quartile cocaine addiction severity	C2	9	-13.10	(-26.07;-0.13)	0.05*	-0.36	(-9.84 ;9.12)	0.94	-11.25	(-44.53 ;22.04)	0.51

*Cocaine addiction severity was categorized by the 3rd quartile; C0=no use, C1 = >0-44% lifetime use, C2 = >44% lifetime use. β: estimated association between BP and both lead and categorical cocaine addiction severity; 95%CI: 95% confidence interval; p: *P* (*P<0.05); BP: blood pressure; MAP: mean arterial pressure: (systolic BP +2 diastolic BP)/3. All results were adjusted for sex (male, female), age (year, continuous), smoking status (yes/no) and education (years, continuous)

Table S2: Cocaine addiction severity[a] modifies the relationship between tibia lead (log$_2$ transformed) and blood pressure levels

Variable	Category	*n*	Systolic BP				Diastolic BP				MAP			
			β	95% CI	*P*	*P* for inter-action	β	95% CI	*P*	*P* for inter-action	β	95% CI	*P*	*P* for inter-action
No cocaine use	C0	15	3.44	(-2.59 ;9.47)	0.26		2.52	(-0.69 ;5.73)	0.12		2.76	(-0.65 ;6.16)	0.11	
<3rd quartile cocaine addiction severity	C1	11	-3.44	(-18.16 ;11.27)	0.65	0.40	1.62	(-6.95 ;10.19)	0.71	0.85	0.31	(-10.14 ;10.75)	0.95	0.67
>3rd quartile cocaine addiction severity	C2	9	17.89	(9.52 ;26.26)	<.0001*	0.01*	10.56	(7.33 ;13.79)	<.0001*	0.003*	13.09	(10.34 ;15.83)	<.0001*	<.0001*

[a]Cocaine addiction severity was categorized by the 3rd quartile; C0=no use, C1 = > 0-44% lifetime use, C2 = >44% lifetime use. β = estimated association between lead exposure and BP in each categorical cocaine addiction severity; 95%CI: 95% Confidence Interval; p: *P*; p-interaction between bone lead and cocaine use on BP, *P<0.05); BP: blood pressure; mean arterial pressure (MAP) = (systolic BP +2 diastolic BP)/3. All results were adjusted for sex (male, female), age (year, continuous), smoking status (yes/no) and education (years, continuous)

Table S3: Secondary Analysis. Multiple drug dependencea modifies the relationship between bone lead levels (log$_2$ transformed ug/g bone) and blood pressure, including repeated measures

Variable	Category	*n*	Systolic BP				Diastolic BP				Mean Arterial Pressure			
			β	95% CI	*P*	*P* for interaction	β	95% CI	*P*	*P* for interaction	β	95% CI	*P*	*P* for interaction
None	D0	10	2.874	(-5.15; 10.9)	0.483	Ref	3.586	(-0.23; 7.41)	0.066	Ref	3.189	(-0.91; 7.29)	0.127	Ref
Cocaine only	D1	6	-11.771	(-28.29; 4.75)	0.163	0.128	-2.111	(-9.92; 5.7)	0.596	0.237	-5.103	(-15.28; 5.07)	0.326	0.149
Multiple drugs	D2	10	14.571	(4.48; 24.66)	0.005	0.064	6.873	(3.66; 10.08)	<.0001	0.183	9.321	(6.46; 12.18)	<.0001	0.012

[a]Multiple drug dependence reflects no drug use (D0), cocaine only (D1) and multiple drugs (D2). β = estimated association between BP with lead exposure in each categorical multiple drug dependence; 95%CI: 95% Confidence Interval; p: *P*; p-interaction between bone lead and multiple drug dependence on BP, *P<0.05); BP: blood pressure; mean arterial pressure (MAP) = (systolic BP +2 diastolic BP)/3. All results were adjusted for sex (male, female), age (year, continuous), smoking status (yes/no) and education (years, continuous).

Table S4: Sensitivity analyses. Main effect of bone lead (log$_2$- transformed ug/g bone) and cocaine addiction severity * on blood pressure levels, including BMI as a covariate and excluding repeated measures (*n*=27)

Variable	Category	*n*	Systolic BP			Diastolic BP			MAP		
			β	95% CI	*P*	β	95% CI	*P*	β	95% CI	*P*
Lead	ug/g bone	27	4.87	(-3.26; 12.99)	0.26	4.87	(0.72; 9.02)	0.03	4.87	(-0.28; 10.01)	0.08
No cocaine use	C0	12	Ref.	.	.	Ref.	.	.	Ref.	.	.
<3rd quartile cocaine addiction severity	C1	10	5.54	(-18.03; 29.1)	0.65	7.37	(-4.66; 19.4)	0.25	6.76	(-8.16; 21.68)	0.39
>3rd quartile cocaine addiction severity	C2	5	-8.49	(-38.26; 21.28)	0.58	2.25	(-12.95; 17.45)	0.78	-1.33	(-20.18; 17.52)	0.89

*Cocaine addiction severity was categorized by the 3rd quartile; C0=no use, C1 = > 0-44% lifetime use, C2 = >44% lifetime use. β: estimated association between BP and both lead and categorical cocaine addiction severity; 95%CI: 95% confidence interval; p: *P* (*P<0.05); BP: blood pressure; MAP: mean arterial pressure: (systolic BP +2 diastolic BP)/3. All results were adjusted for sex (male, female), age (year, continuous), smoking status (yes/no) and education (years, continuous)

Table S5: Sensitivity analyses. Cocaine addiction severity a modifies the relationship between bone lead (log$_2$ transformed ug/g bone) and blood pressure levels when including BMI as a covariate and excluding repeated measures (*n*=26)

Variable	Category	*n*	Systolic BP				Diastolic BP				MAP			
			β	95% CI	*P*	*P* for interaction	β	95% CI	*P*	*P* for interaction	β	95% CI	*P*	*P* for interaction
No cocaine use	C0	12	2.95	(-9.2; 15.11)	0.64		2.68	(-3.35; 8.7)	0.40		2.77	(-4.76; 10.29)	0.48	
<3rd quartile cocaine addiction severity	C1	10	-3.91	(-23.38; 15.57)	0.70	0.58	1.24	(-8.42; 10.9)	0.80	0.81	-0.47	(-12.53; 11.58)	0.94	0.67
>3rd quartile cocaine addiction severity	C2	5	13.76	(-2.21; 29.72)	0.11	0.33	10.88	(2.96; 18.79)	0.02	0.14	11.84	(1.95; 21.72)	0.03	0.19

[a]Cocaine addiction severity was categorized by the 3rd quartile; C0=no use, C1 = > 0-44% lifetime use, C2 = >44% lifetime use. β = estimated association between lead exposure and BP in each categorical cocaine addiction severity; 95%CI: 95% Confidence Interval; p: *P*; p-interaction between bone lead and cocaine use on BP, *P<0.05); BP: blood pressure; mean arterial pressure (MAP) = (systolic BP+2 diastolic BP)/3. All results were adjusted for sex (male, female), age (year, continuous), smoking status (yes/no) and education (years, continuous).

[Downloaded free from http://www.environmentmed.org on Monday, October 14, 2019, IP: 146.203.130.213]

Table S6: Main effect of bone lead concentrations (log_2 transformed ug/g bone) or cocaine addiction severity* on blood pressure levels. All models were adjusted for the inverse probability of being a CUD participant, computed using age, smoking, education and lead exposure

Variable	Category	*n*	Systolic BP			Diastolic BP			MAP		
			β	95% CI	*P*	β	95% CI	*P*	β	95% CI	*P*
Lead	mg/g bone	35	6.461	(1.44; 11.48)	0.012	5.187	(1.52; 8.85)	0.006	5.576	(1.61; 9.54)	0.006
No cocaine use	C0	15									
<3rd quartile cocaine addiction severity	C1	11	10.309	(-1.39; 22)	0.084	3.364	(-4.71; 11.44)	0.414	5.631	(-2.3; 13.56)	0.164
>3rd quartile cocaine addiction severity	C2	9	-9.736	(-19.94; 0.47)	0.062	-0.876	(-7.59; 5.84)	0.798	-3.910	(-11.24; 3.42)	0.296

*Cocaine addiction severity was categorized by the 3rd quartile; C0=no use, C1 = >0-44% lifetime use, C2 = >44% lifetime use. β: estimated association between BP and both lead and categorical cocaine addiction severity; 95%CI: 95% confidence interval; p: *P* (*P<0.05); BP: blood pressure; MAP: mean arterial pressure: (systolic BP +2 diastolic BP)/3. All results were adjusted for sex (male, female), age (year, continuous), smoking status (yes/no) and education (years, continuous)

Table S7: Cocaine addiction severitya modifies the relationship between tibia lead (log_2 transformed) and blood pressure levels. All models were adjusted for the inverse probability of being a CUD participant, computed using age, smoking, education and lead exposure.

Variable	Category	*n*	Systolic BP				Diastolic BP				MAP			
			β	95% CI	*P*	*P* for interaction	β	95% CI	*P*	*P* for interaction	β	95% CI	*P*	*P* for interaction
No cocaine use	C0	15	2.798	(-2.54; 8.14)	0.304		2.550	(-0.84; 5.94)	0.140		2.690	(-1.09; 6.47)	0.163	
<3rd quartile cocaine addiction severity	C1	11	-2.504	(-17.8; 12.79)	0.748	0.527	4.117	(-5.9; 14.13)	0.421	0.772	2.047	(-9.39; 13.48)	0.726	0.916
>3rd quartile cocaine addiction severity	C2	9	17.766	(9.26; 26.27)	<.0001	0.009	10.718	(7.56; 13.87)	<.0001	0.003	13.139	(10.29; 15.99)	<.0001	0.0004

[a]Cocaine addiction severity was categorized by the 3rd quartile; C0=no use, C1 = > 0-44% lifetime use, C2 = >44% lifetime use. β = estimated association between lead exposure and BP in each categorical cocaine addiction severity; 95%CI: 95% Confidence Interval; p: p; p-interaction between bone lead and cocaine use on BP, *P<0.05); BP: blood pressure; mean arterial pressure (MAP) = (systolic BP +2 diastolic BP)/3. All results were adjusted for sex (male, female), age (year, continuous), smoking status (yes/no) and education (years, continuous).

Table S8: Main effect of bone lead concentrations (log_2 transformed ug/g bone) or cocaine addiction severity* on blood pressure levels in men only

Variable	Category	*n*	Systolic BP			Diastolic BP			MAP		
			β	95% CI	*P*	β	95% CI	*P*	β	95% CI	*P*
Lead	mg/g bone	35	6.663	(-0.09; 13.42)	0.053	5.527	(1.49; 9.57)	0.007	5.865	(1.33; 10.4)	0.011
No cocaine use	C0	15									
<3rd quartile cocaine addiction severity	C1	11	-0.394	(-19.58; 18.79)	0.968	0.688	(-13.49; 14.87)	0.924	1.283	(-12.36; 14.92)	0.854
>3rd quartile cocaine addiction severity	C2	9	-2.003	(-21.13; 17.13)	0.838	0.521	(-12.81; 13.85)	0.939	-3.134	(-15.83; 9.56)	0.628

*Cocaine addiction severity was categorized by the 3rd quartile; C0=no use, C1 = >0-44% lifetime use, C2 = >44% lifetime use. β: estimated association between BP and both lead and categorical cocaine addiction severity; 95%CI: 95% confidence interval; p: *P* (*P<0.05); BP: blood pressure; MAP: mean arterial pressure: (systolic BP +2 diastolic BP)/3. All results were adjusted for sex (male, female), age (year, continuous), smoking status (yes/no) and education (years, continuous)

Table S9: Cocaine addiction severity[a] modifies the relationship between tibia lead (log_2 transformed) and blood pressure levels in men only

Variable	Category	*n*	Systolic BP				Diastolic BP				MAP			
			β	95% CI	*P*	*P* for interaction	β	95% CI	*P*	*P* for interaction	β	95% CI	*P*	*P* for interaction
No cocaine use	C0	15	3.704	(-3.04; 10.45)	0.282		4.191	(-0.71; 9.1)	0.094		3.737	(-0.58; 8.06)	0.090	
<3rd quartile cocaine addiction severity	C1	11	-5.895	(-17.87; 6.08)	0.335	0.182	-0.167	(-8.18; 7.85)	0.967	0.403	-1.624	(-10.92; 7.67)	0.732	0.328
>3rd quartile cocaine addiction severity	C2	9	17.425	(8.99; 25.86)	<.0001	0.024	10.269	(7.02; 13.52)	<.0001	0.068	12.716	(9.71; 15.73)	<.0001	0.002

[a]Cocaine addiction severity was categorized by the 3rd quartile; C0=no use, C1 = > 0-44% lifetime use, C2 = >44% lifetime use. β = estimated association between lead exposure and BP in each categorical cocaine addiction severity; 95%CI: 95% Confidence Interval; p: *P*; p-interaction between bone lead and cocaine use on BP, *P<0.05); BP: blood pressure; mean arterial pressure (MAP) = (systolic BP +2 diastolic BP)/3. All results were adjusted for sex (male, female), age (year, continuous), smoking status (yes/no) and education (years, continuous).
